# The impact of anthracyclines in intermediate and high-risk HER2-negative early breast cancer—a pooled analysis of the randomised clinical trials PlanB and SUCCESS C

**DOI:** 10.1038/s41416-021-01690-6

**Published:** 2022-02-22

**Authors:** Amelie de Gregorio, Wolfgang Janni, Thomas W. P. Friedl, Ulrike Nitz, Brigitte Rack, Andreas Schneeweiss, Ronald Kates, Tanja Fehm, Hans Kreipe, Matthias Christgen, Sherko Kümmel, Elisabeth Trapp, Rachel Wuerstlein, Andreas Hartkopf, Michael Clemens, Toralf Reimer, Lothar Häberle, Peter A. Fasching, Oleg Gluz, Nadia Harbeck

**Affiliations:** 1 Department of Gynecology and Obstetrics, Ulm University Hospital, Ulm, Germany; 2 Department of Gynecology and Obstetrics, Evangelical Hospital Bethesda, Moenchengladbach, Germany; 3 Westdeutsche Studiengruppe (WSG), Moenchengladbach, Germany; 4 National Center for Tumor Diseases, University Hospital and German Cancer Research Center, Heidelberg, Germany; 5 Department of Gynecology and Obstetrics, Duesseldorf University Hospital, Heinrich-Heine University, Duesseldorf, Germany; 6 Institute of Pathology, Medical School Hannover, Hannover, Germany; 7 Breast Unit, Kliniken Essen-Mitte, Essen, Germany; 8 Clinic for Gynecology with Breast Center, Charité-Universitaetsmedizin Berlin, Berlin, Germany; 9 Department of Gynecology and Obstetrics, University of Graz, Graz, Austria; 10 Breast Center, Department of Gynecology and Obstetrics and CCC Munich, LMU University Hospital, Munich, Germany; 11 Department of Gynecology and Obstetrics, Tuebingen University Hospital, Tuebingen, Germany; 12 Mutterhaus der Borromaerinnen, Trier, Germany; 13 Department of Obstetrics and Gynecology, University of Rostock, Rostock, Germany; 14 Department of Gynecology and Obstetrics, Erlangen University Hospital, Friedrich-Alexander-University of Erlangen-Nuremberg, Comprehensive Cancer Center EMN, Erlangen, Germany

**Keywords:** Randomized controlled trials, Breast cancer

## Abstract

**Background:**

Anthracycline/cyclophosphamide-taxane-containing chemotherapy (AC-T) is the standard of care in the adjuvant treatment of HER2-negative early breast cancer (EBC), but recent studies suggest omission of anthracyclines for reduced toxicity without compromising efficacy.

**Methods:**

Based on individual patient data (*n* = 5924) pooled from the randomised Phase III trials PlanB and SUCCESS C, we compared disease-free survival (DFS) and overall survival (OS) between intermediate to high-risk HER2-negative EBC-patients treated with either six cycles of docetaxel/cyclophosphamide (TC6) or an AC-T regime using univariable and adjusted multivariable Cox regression models.

**Results:**

AC-T conferred no significant DFS or OS advantage in univariable (DFS: hazard ratio (HR) for TC vs. AT 1.05, 95% confidence interval (CI): 0.89–1.24, *P* = 0.57; OS: HR 1.00, 95% CI: 0.80–1.26, *P* = 1.00) and adjusted multivariable analysis (DFS: HR 1.01, 95% CI: 0.86–1.19, *P* = 0.91; OS: HR 0.97, 95% CI: 0.77–1.22, *P* = 0.79). Patients receiving TC6 had significantly fewer grade 3–4 adverse events. Exploratory subgroup analysis showed that AC-T was associated with significantly better DFS and OS in pN2/3 patients, specifically in those with lobular histology.

**Conclusion:**

For most patients with HER2-negative EBC, AC-T is not associated with a survival benefit compared to TC6. However, patients with lobular pN2/pN3 tumours seem to benefit from anthracycline-containing chemotherapy.

## Introduction

Current international guidelines recommend anthracycline/taxane-based chemotherapy as the standard cytotoxic regime for human epidermal growth factor receptor 2 (HER2)-negative early breast cancer (EBC) [1]. Anthracycline-free regimes are currently restricted to patients unwilling or unable to be treated with anthracyclines, e.g., because of cardiac comorbidities or in patients with lower-risk disease.

In 2003, Henderson et al. showed that the addition of sequential paclitaxel to the adjuvant anthracycline-containing chemotherapy (AC) improved outcomes in patients with node-positive primary breast cancer (5-year disease-free survival (DFS) rate for AC plus paclitaxel 70% vs 65% for AC, 5-year overall survival (OS) rate 80% vs 77%). However, no benefit was seen with escalation of doxorubicin dose [2]. These findings were supported by the results of the randomised PACS01 trial in 2006, where 1999 patients with node-positive early breast cancer were treated with either six cycles of FEC (fluorouracil, epirubicin, and cyclophosphamide) or three cycles of FEC followed by three cycles of docetaxel. The outcome was better in the taxane-containing treatment group with a 5-year DFS rate of 78.4% vs 73.2% and a 5-year OS rate of 90.7% vs 86.7% [3].

A meta-analysis from the Early Breast Cancer Trialists’ Collaborative Group (EBCTCG) compared the long-term outcome of 123 randomised trials for women with early breast cancer based on different polychemotherapeutic regimes. Based on a cohort of 11.167 patients, the effect of adding four cycles of taxanes to anthracyclines (experimental arm with prolonged treatment duration) was compared to the standard arm with anthracycline-containing chemotherapy only. The 8-year recurrence rate was 30.2% for the taxane-group vs 34.8% for the anthracycline-control arm (RR 0.84; 96% CI: 0.78–0.91), the 8-year breast cancer mortality was 21.1% vs 23.9% (RR 0.86; 95% CI: 0.79–0.93) and 8-year overall mortality was 23.5% vs 26.7% (RR 0.86; 95% CI: 0.79–0.93). Based on additional findings of their large meta-analysis, the authors concluded that the 10-year risk of breast cancer mortality can be reduced by on average about one-third with taxane/anthracycline-based chemotherapy regimens or higher-cumulative-dosage anthracycline-based regimens compared to no chemotherapy [4].

Taxane-containing anthracycline-free adjuvant chemotherapy offers an additional evidence-based therapy option for selected early breast cancer patients. Jones et. reported in 2006 the first results of a randomised Phase III trial comparing four cycles of TC (docetaxel cyclophosphamide) or four cycles of standard-dose AC in more than 1000 early breast cancer patients. Both 5-year DFS rate and 5-year OS rate were significantly higher for TC than for AC (86% vs 80 and 90% vs 87%, respectively) [5]. These findings were later confirmed based on even longer follow-up (DFS 81% TC vs 75% AC and OS 87% TC vs 82% AC) [6], allowing to offer an anthracycline-free less cytotoxic therapy regimen to lower-risk patients. Nevertheless, the impact of anthracyclines needs to be addressed not only for low-risk but also for high-risk early breast cancer patients for avoiding unnecessary severe long-term toxicities through anthracyclines like acute myeloid leukaemia or cardiotoxicity [7, 8].

In 2017, Blum et al. presented a joint efficacy analysis from the three adjuvant ABC trials (US Oncology Research (USOR) 06-090, National Surgical Adjuvant Breast and Bowel Project (NSABP) B-46-I/USOR 07132 and NSABP B-49) and concluded that anthracycline/taxane-based chemotherapy improved invasive DFS in patients with high-risk HER2-negative breast cancer compared to TC6 because the obtained hazard ratio exceeded the prespecified inferiority boundary [9]. Based on current knowledge, taxanes should be considered as therapy backbone in standard cytotoxic treatment of early breast cancer and thus, further analyses evaluating the impact of anthracyclines for adjuvant chemotherapy in high-risk HER2-negative early breast cancer patients have to come from taxane-treated patient cohorts. We, therefore, compared the outcome of high-risk early breast cancer patients treated either with six cycles of TC or sequential anthracycline/taxane chemotherapy using a large pooled set of individual patient data from two randomised Phase III clinical trials, PlanB and SUCCESS C. Both studies were originally designed as separate non-inferiority trials. The results of PlanB have already been published, showing that TC6 was equally effective as sequential anthracycline/taxane chemotherapy in HER2-negative early breast cancer [10]. Results of the similarly designed SUCCESS C trial have not been published before and are first presented as part of this—not preplanned—pooled analysis, which was conducted to increase the statistical power and facilitate exploratory analyses of the effects of anthracycline-containing vs anthracycline-free chemotherapy in clinically meaningful subgroups.

## Materials and methods

### Study designs

Individual patient data from the two German prospective multicenter randomised Phase III trials “PlanB” (NCT01049425, 91 centres) and “Success C” (NCT00847444, 231 centres) were combined for a pooled analysis. In both trials, patients were assigned to an anthracycline-free treatment cohort (experimental arm “A-free”, consisting of six cycles of TC6) or an anthracycline-containing chemotherapy arm (“A-containing”; AC-T), based on the initially administered chemotherapy regime.

For both PlanB and Success C, the experimental A-free chemotherapy arm consisted of six cycles of docetaxel 75 mg/m² and cyclophosphamide 600 mg/m² (TC6) every 3 weeks. The standard treatment A-containing arm in PlanB consisted of four cycles of epirubicin 90 mg/m² and cyclophosphamide 600 mg/m² followed by four cycles of docetaxel 100 mg/m² every 3 weeks (EC-DOC). The A-containing standard treatment arm of the Success C trial comprised three cycles of 5-fluorouracil 500 mg/m², epirubicin 100 mg/m² and cyclophosphamide 500 mg/m² followed by three cycles of docetaxel 100 mg/m² every 3 weeks (FEC-DOC) [11].

Adjuvant therapy with radiotherapy and/or endocrine treatment was allowed in both studies according to the national guideline recommendations. The primary endpoint of both studies was the comparison of DFS between both treatment arms. DFS was defined as time interval without any invasive local, regional or distant disease recurrence. Secondary endpoints included OS and safety. Toxicities were regularly assessed during treatment and afterwards. Follow-up examinations were performed according to national guidelines over at least 5 years. In both studies, various translational research programs complemented the clinical trials [12, 13].

### Patients

Within the PlanB trial, 3198 patients were recruited between 2009 and 2011, and the Success C study enrolled 3643 patients from 2008 to 2011. All randomised patients were ≥18 years and fulfilled the defined inclusion criteria. In both studies, patients with high-risk, HER2-negative invasive early breast cancer and no evidence of distant metastases were eligible after completion of local breast cancer therapy with complete resection of the tumour and axillary surgery.

In the PlanB study, the definition of high-risk patients included women with node-positive early breast cancer or node-negative tumours and additional risk factors like tumours ≥T2-Tumours, histopathological grade (G) 2/3, high urokinase plasminogen activator/plasminogen activator inhibitor-1 (uPA/PAI-1), negative hormone-receptor (HR) status or age ≤35 years. The histological type was reviewed by central pathology [14]. After an early amendment, prospective genomic testing by OncotypeDX was offered to all patients with HR + /HER2− disease with 0–3 positive lymph nodes and omission of chemotherapy were allowed with a Recurrence Score ≤11. For PlanB, *n* = 3198 were screened and *n* = 2449 randomised. Only patients with chemotherapy treatment and available follow-up (*n* = 2281) were included in the pooled analysis. For Success C, high-risk early breast cancer was similarly defined as positive axillary lymph nodes or women with node-negative disease but at least pT2-Tumours or histopathological grade 3, age ≤35 or negative hormone-receptor status. All 3463 patients randomised for the Success C trial were included in the pooled analysis.

Patients with inflammatory breast cancer, severe comorbidities or relevant cardiac disorders were not eligible for both trials. All participating patients provided written informed consent. Both trials were approved by the German ethics boards and conducted in accordance to the Declaration of Helsinki.

Adverse events were assessed according to the National Cancer Institute Common Toxicity Criteria (CTCAE) version 3.0 and were collected after each cycle of chemotherapy and 3 to 4 weeks after the last chemotherapy treatment.

Biological subtypes were defined based on hormone-receptor status and histopathological grade as follows: “luminal A-like” tumours were defined as HR-positive, G1/2, “luminal B-like” tumours as hormone receptor-positive, G3, and triple-negative (TN) tumours as hormone-receptor-negative, HER2-negative (HER2-negative primary tumour was a mandatory inclusion criterion in both studies).

### Statistical analysis

Both studies were originally designed as non-inferiority trials, albeit with different non-inferiority margins. For PlanB, the hazard ratio based non-inferiority margin was equivalent to a 4.4% difference in 5-year DFS, while the non-inferiority margin for SUCCESS C was set to a 4.0% difference in 5-year DFS. However, the pooled analysis was not primarily intended and there was no prospective statistical plan for the analysis of the combined dataset; thus, the analysis of the pooled data was not performed according to an inferiority trial design. The pooled analysis had a (retrospectively calculated) power of 80% to detect a 2.0% difference in 5-year DFS, equivalent to a hazard ratio of 1.21 (two-sided test with *α* = 0.05).

Patient outcomes in terms of DFS and OS were analysed following the standardised definitions for efficacy endpoints (STEEP) criteria [15]. Accordingly, DFS was defined as the time from randomisation to the earliest date of disease progression (any invasive ipsilateral, regional, contralateral, and distant disease recurrence, second primary tumours, or death from any cause; non-invasive, in-situ cancer events were excluded) or the date of censoring. OS was defined accordingly with death from any cause as an event. Survival rates based on time-to-event data were estimated by the Kaplan–Meier product-limit method and survival curves were compared using log-rank tests. Hazard ratios (HRs) with 95% confidence intervals were estimated using univariable and multivariable Cox models adjusted for hormone-receptor (HR) status (negative, positive), histological grade (G1, G2, G3), age (≤40, 41–60, >60), menopausal status (premenopausal, postmenopausal), type of surgery (breast conserving, mastectomy, other), pT (pT1, pT2, pT3/pT4), pN (pN0/pN1, pN2/pN3), histological type (ductal, lobular, other), and study (Success C, PlanB). The two-way interactions between the chemotherapy treatment arm and each of the different factors presented for all of the exploratory subgroup analyses (see forest plots) were calculated using a Cox regression model with the two main effects and the corresponding two-way interaction (not adjusted for other factors). In two cases, we explored the relationships between the chemotherapy treatment arm and two of the other factors simultaneously in more detail by calculating three-way interactions (chemotherapy treatment arm * nodal status * HR status; chemotherapy treatment arm * nodal status * histological type; see “Results”). This was done to more specifically identify clinically meaningful patient subgroups that might benefit from A-containing chemotherapy in cases where the results of the two-way interactions warranted further investigations. To achieve this, we used adjusted cox regression models that included all main effects, the two two-way interactions between the chemotherapy treatment arm and both of the two other factors and the three-way interaction. We did not implement forward, backward or stepwise selection procedures and results of the full models only are reported here.

## Results

### Baseline characteristics

**Fig. 1:**
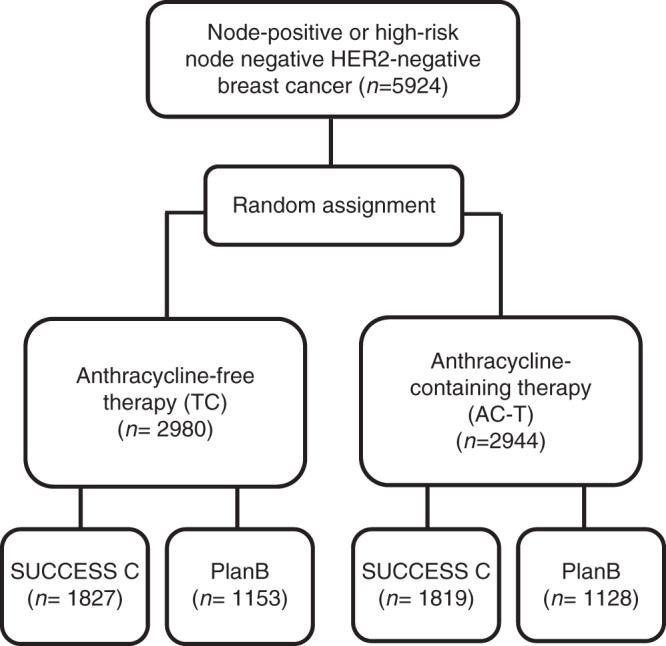
Consort Diagram. CONSORT patient flow diagram of the PlanB and Success C pooled analysis.

For this pooled analysis, data from 5924 patients (2281 patients in PlanB and 3643 in Success C) with follow-up were available. In total, 2980 patients were randomised to TC6 and 2944 patients to AC-T (see Fig. 1 for CONSORT diagram). Median follow-up was 62.0 months (60.0 months PlanB, 64.6 months Success C). The median age was 55 years, 51.7% of patients had nodal-positive and 21.6% HR-negative disease. Baseline patient and tumour characteristics were well-balanced between the two chemotherapy treatment arms of this pooled analysis (Table 1). Detailed information on patient and tumour characteristics according to trial (Success C, PlanB) is provided in Supplementary Table S1.

**Table 1:** Baseline and clinic-pathological characteristics of patients according to chemotherapy arm.

Variable	Total *N* = 5924	Anthracycline-containing chemotherapy^a^, *N* = 2944	Anthracycline-free chemotherapy^b^, *N* = 2980	*P* value^c^
Study				0.766^d^
Success C	3643 (61.5%)	1816 (61.7%)	1827 (61.3%)	
PlanB	2281 (38.5%)	1128 (38.3%)	1153 (38.7%)	
Age (years)				0.675^e^
Median	55.0	55.0	55.0	
Range	24–79	25–78	24–79	
Menopausal status				0.795^d^
Premenopausal	2249 (38.0)	1115 (37.9%)	1134 (38.1%)	
Postmenopausal	3503 (59.1%)	1749 (59.4%)	1754 (58.9%)	
Unknown	172 (2.9%)	80 (2.7%)	92 (3.1%)	
Tumour stage				0.191^f^
pT1	2857 (48.2%)	1456 (49.5%)	1401 (47.0%)	
pT2	2753 (46.5%)	1330 (45.2%)	1423 (47.8%)	
pT3	251 (4.2%)	124 (4.2%)	127 (4.3%)	
pT4	63 (1.1%)	34 (1.2%)	29 (1.0%)	
Nodal stage				0.938^f^
pN0	2859 (48.3%)	1414 (48.0%)	1445 (48.5%)	
pN1	2485 (41.9%)	1246 (42.3%)	1239 (41.6%)	
pN2	430 (7.3%)	211 (7.2%)	219 (7.3%)	
pN3	149 (2.5%)	73 (2.5%)	76 (2.6%)	
Unknown	1 (0.0%)	0 (0.0%)	1 (0.0%)	
Histological grading				0.805^f^
G1	369 (6.2%)	184 (6.3%)	185 (6.2%)	
G2	3167 (53.5%)	1567 (53.2%)	1600 (53.7%)	
G3	2384 (40.2%)	1191 (40.5%)	1193 (40.0%)	
Unknown	4 (0.1%)	2 (0.1%)	2 (0.1%)	
Histological type				0.554^d^
Ductal	4808 (81.2%)	2393 (81.3%)	2415 (81.0%)	
Lobular	726 (12.3%)	350 (11.9%)	376 (12.6%)	
Other	390 (6.6%)	201 (6.8%)	189 (6.3%)	
Hormone-receptor status				0.869^d^
Negative	1279 (21.6%)	633 (21.5%)	646 (21.7%)	
Positive	4645 (78.4%)	2311 (78.5%)	2334 (78.3%)	
Type of surgery				0.755^d^
Breast conserving	4642 (78.4%)	2313 (78.6%)	2329 (78.2%)	
Mastectomy	1141 (19.3%)	558 (19.0%)	583 (19.6%)	
Other	141 (2.4%)	73 (2.5%)	68 (2.3%)	
Biological subtype				0.968^d^
Luminal A-like	3323 (56.1%)	1656 (56.3%)	1667 (55.9%)	
Luminal B-like	1319 (22.3%)	653 (22.2%)	666 (22.3%)	
Triple-negative	1279 (21.6%)	633 (21.5%)	646 (21.7%)	
Unknown	3 (0.1%)	2 (0.1%)	1 (0.0%)	
Radiotherapy				0.345^d^
No	1139 (19.2%)	552 (18.8%)	587 (19.7%)	
Yes	4780 (80.7%)	2391 (81.2%)	2389 (80.2%)	
Unknown	5 (0.1%)	1 (0.0%)	4 (0.1%)	

^a^Success C: FEC-Doc; 3 × fluorouracil_500_-epirubicin_100_-cyclophosphamide_500_ q3w followed by 3 × docetaxel_100_ q3w; PlanB: EC-Doc; 4 × epirubicin_90_-cyclophosphamide_600_ q3w followed by 4 × docetaxel_100_ q3w.

^b^Success C and PlanB: Doc-C; 6 × docetaxel_75_-cyclophosphamide_600_ q3w.

^c^All tests without unknowns.

^d^Chi-square test.

^e^Mann–Whitney *U* test.

^f^Cochran–Armitage test for trend.

### Outcome

**Fig. 2:**
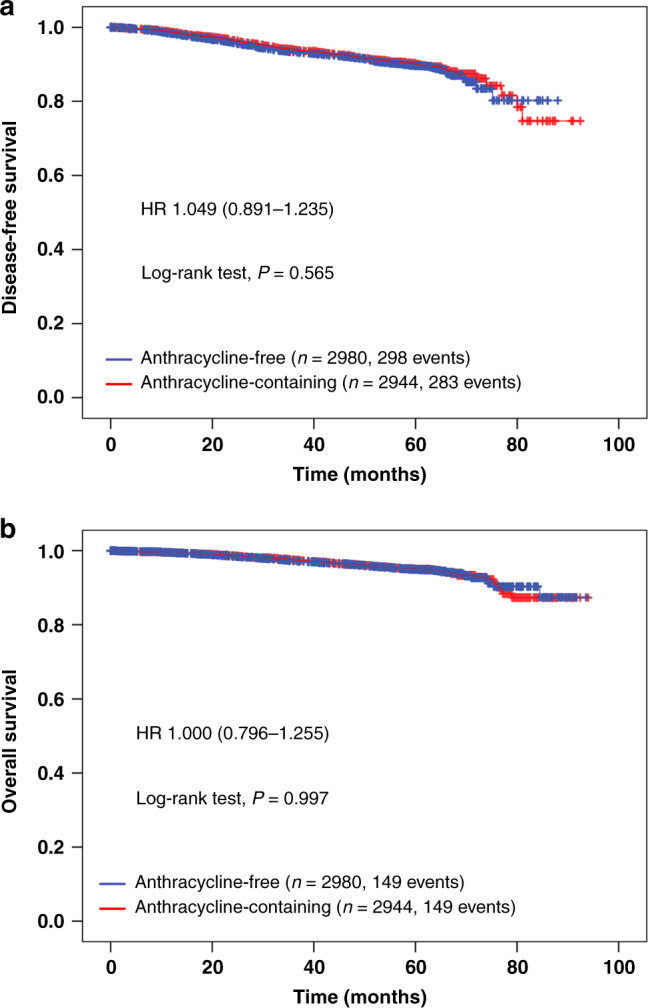
Kaplan-Meier plots showing outcome for anthracycline-free vs. anthracycline-containing chemotherapy. Kaplan–Meier plots for disease-free survival (**a**) and overall survival (**b**) according to chemotherapy arm (anthracycline-free vs. anthracycline-containing) of the PlanB and Success C trials (data pooled).

DFS of patients receiving A-free vs A-containing chemotherapy was quite similar in univariable analysis (hazard ratio, HR = 1.05; 95% confidence interval, CI: 0.89–1.24, *P* = 0.57; Fig. 2a) and in multivariable analysis (HR = 1.01, 95% CI: 0.86–1.19, *P* = 0.91; see Supplementary Table S2). Five-year DFS rate was 90.0% (283 events) in the A-containing and 89.3% (298 events) in the A-free group (see Supplementary Table S3 for a detailed list of DFS events). Similarly, OS did not differ between the A-free vs A-containing study arms in univariable (HR = 1.00; 95% confidence interval, CI: 0.80–1.26, *P* = 1.00; Fig. 2b) and adjusted multivariable analysis (HR = 0.97, 95% CI: 0.77–1.22, *P* = 0.79); 5-year OS rates were 94.9% (149 events) and 95.0% (149 events) in the A-free and A-containing treatment arms, respectively (Fig. 2b).

**Fig. 3:**
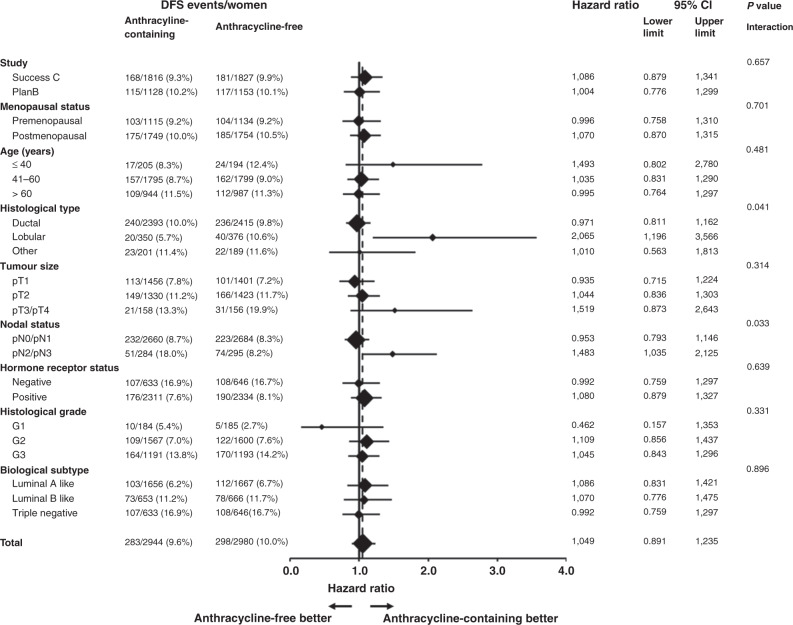
Forest plot showing disease-free survival with anthracycline-free or anthracycline-containing chemotherapy according to the different patient and tumour characteristic subgroups. Forest plot showing results of explorative subgroup analyses in terms of the comparison of disease-free survival between patients with anthracycline-free or anthracycline-containing chemotherapy according to the different patient and tumour characteristic subgroups. The diamonds indicate the hazard ratios (anthracycline-free vs. anthracycline-containing chemotherapy), and diamond size is proportional to the number of patients per subgroup. The horizontal lines indicate the corresponding 95% confidence intervals for the hazard ratios. The solid vertical line represents a hazard ratio of 1.0 (i.e., no difference in survival between anthracycline-free or anthracycline-containing chemotherapy), and the dashed vertical line represents the hazard ratio for the overall analysis with all 5924 patients (hazard ratio 1.049).

The forest plots for the comparison between patients receiving anthracycline-free or anthracycline-containing chemotherapy for all subgroups tested including the *P* values for the two-way interactions are shown in Fig. 3 for DFS (details on OS are presented in the supplement). There were no significant differences in DFS between A-free and A-containing chemotherapy in any of the three different biological tumour subtypes (luminal A-like: HR = 1.09, 95% CI: 0.83–1.42, *P* = 0.55; luminal B-like: HR = 1.07, 95% CI: 0.78–1.48, *P* = 0.68; triple-negative: HR = 0.99, 95% CI: 0.76–1.30, *P* = 0.96). Similar results with no differences between A-free and A-containing chemotherapy were obtained for OS (luminal A-like: HR = 0.94, 95% CI: 0.64–1.39, *P* = 0.76; luminal B-like: HR = 0.92, 95% CI: 0.57–1.48, *P* = 0.72; triple-negative: HR = 1.08, 95% CI: 0.76–1.53, *P* = 0.66). Furthermore, there were no significant two-way interactions between chemotherapy treatment arm (A-free or A-containing) and biological subtype for DFS (*P* = 0.896) or OS (*P* = 0.828), indicating that there is no evidence for a survival benefit of either A-containing or A-free chemotherapy depending on biological subtype. Similar results with no significant two-way interactions with respect to both DFS and OS were obtained with regard to subgroups defined by study (PlanB, Success C), menopausal status (premenopausal, postmenopausal), age (≤40 years, 41–60 years, >60 years), tumour size (pT1, pT2, pT3/pT4), HR status (negative, positive), and histological grade (G1, G2, G3).

No difference in DFS between the two chemotherapy arms was found for patients with pN0/pN1 tumours (HR = 0.95, 95% CI: 0.79–1.15, *P* = 0.61; Supplementary Fig. S1A). In contrast, a significant difference between TC6 and AC-T in the subgroup analyses was found for patients with more than three affected lymph nodes (pN2/pN3 tumours), who had a significantly worse DFS when treated with A-free chemotherapy (HR = 1.48, 95% CI: 1.04–2.13, *P* = 0.031; Supplementary Fig. S1B). The resulting significant two-way interaction (*P* = 0.033) between chemotherapy arm (TC6 or AC-T) and nodal status (pN0/pN1 or pN2/pN3) suggests that the effect of AC-T depends on the extent of lymph node involvement, with patients that have pN2 or pN3 tumours benefitting from A-containing chemotherapy. Further analyses showed that the two-way interaction between chemotherapy arm (TC6 or AC-T) and nodal status (pN0/pN1 or pN2/pN3) was significant in HR-positive tumours (*P* = 0.019) but not in triple-negative tumours (*P* = 0.89), indicating that the benefit from A-containing chemotherapy in patients with pN2/pN3 tumours seems to be restricted to luminal-like tumours only. To formally test whether the effect of nodal status on the efficacy of AC-T depends on hormone-receptor status, we performed an adjusted multivariable cox regression model (including all main effects, the two-way interactions between chemotherapy regime and nodal status and between chemotherapy regime and hormone-receptor status, and the three-way interaction between chemotherapy arm, nodal status and hormone receptor status). However, this model revealed no significant three-way interaction between chemotherapy arm, nodal status and hormone-receptor status (*P* = 0.245), indicating that the influence of nodal status on the efficacy of AC-T was not significantly modulated by hormone-receptor status.

The only other significant difference between A-free and A-containing chemotherapy was obtained in the subgroup analysis for lobular cancer. Patients with lobular carcinomas receiving TC6 had significantly worse DFS compared to patients with lobular carcinomas receiving A-containing chemotherapy (HR = 2.07, 95% CI: 1.20–3.57, *P* = 0.008; Supplementary Fig. S2B). No difference in DFS between the two chemotherapy regimens was found for patients with ductal carcinomas (HR = 0.97, 95% CI: 0.81–1.16, *P* = 0.75; Supplementary Fig. S2A) or patients with carcinomas of a histological type other than ductal or lobular (HR = 1.01, 95% CI: 0.56–1.81, *P* = 0.97; Supplementary Fig. S2C). Furthermore, the two-way interaction between chemotherapy treatment arm and histological type was significant (*P* = 0.041), indicating that patients with lobular carcinomas—in contrast to patients with other tumour histology—show better DFS with A-containing chemotherapy.

We also performed an exploratory analysis to investigate whether the effect of anthracycline-containing chemotherapy observed in patients with lobular carcinomas was dependent on menopausal status. However, there was no significant two-way interaction between chemotherapy treatment arm and menopausal status (*P* = 0.091), indicating that the benefit of anthracycline-containing chemotherapy in patients with lobular carcinomas was not affected by menopausal status. More details and results of this exploratory analysis are presented in the Supplementary Material (Supplementary Material S1).

**Fig. 4:**
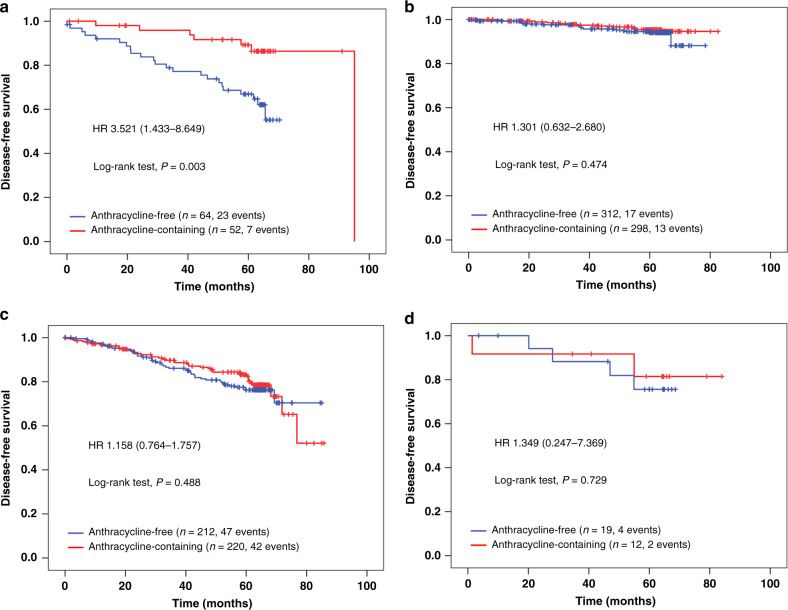
Kaplan-Meier showing disease-free survival in different subgroups according to chemotherapy arm. Kaplan–Meier plots for disease-free survival in different subgroups according to chemotherapy arm (anthracycline-free vs. anthracycline-containing) of the PlanB and Success C trials (data pooled) for patients with pN2/pN3 lobular tumours (**a**), pN0/pN1 lobular tumours (**b**), pN2/pN3 ductal tumours (**c**), and pN2/pN3 tumours with another histological type (**d**). In addition to the results of the log-rank test, univariable hazard ratios and 95% confidence intervals are shown.

Taken together, our data (i.e. the significant two-way interactions) suggest that both patients with lobular tumours and patients with pN2/pN3 tumours might benefit from A-containing chemotherapy in terms of longer DFS. To explore these effects in more detail, we used an adjusted multivariable cox regression model including all main effects, the two-way interactions between chemotherapy treatment arm and both nodal status and histological type, and the three-way interaction between chemotherapy arm, nodal status and histological type. In this model, the two-way interactions between chemotherapy treatment arm * nodal status and chemotherapy treatment arm * histological type became non-significant (*P* = 0.688 and *P* = 0.746, respectively), while the three-way interaction was significant (*P* = 0.028; see Supplementary Table S4). This result indicates that the benefit of A-containing chemotherapy was not evident in all patients with pN2/pN3 tumours and in all patients with lobular carcinomas, but was only seen in patients that had both, i.e. a lobular tumour with more than three affected lymph nodes (HR 3.52, 95% CI: 1.43–8.65; Fig. 4a), with a 5-year DFS rate of 89.0% in the A-containing and 65.8% in the A-free group. In contrast, there were no significant differences in DFS between A-containing and A-free chemotherapy for patients with pN0/pN1 lobular carcinomas (HR 1.30, 95% CI: 0.63–2.68; Fig. 4b), for patients with pN2/pN3 ductal carcinomas (HR 1.16, 95% CI: 0.76–1.76; Fig. 4c) and for patients with pN2/pN3 carcinomas with histological types other than ductal or lobular (HR 1.35, 95% CI: 0.25–7.37, Fig. 4d).

There were no significant two-way interactions between the chemotherapy arm (anthracycline-free or anthracycline-containing) and any of the subgroup variables tested with regard to OS (see Supplementary Fig. S3). However, similar to DFS, an adjusted multivariable cox regression model including all main effects, the two-way interactions between chemotherapy treatment arm and both nodal status and histological type, and the three-way interaction between chemotherapy arm, nodal status and histological type yielded a significant three-way interaction (*P* = 0.022), confirming an OS benefit for patients with lobular pN2/pN3 tumours (univariable HR 3.41, 95% CI: 1.10–10.56, *P* = 0.034; see Supplementary Fig. S4).

### Adverse events

Table 2 lists the toxicity summary from PlanB and Success C with the description of well-known adverse events. During the course of the Success C study, 24 unexpected serious events (SUSARs) were observed; one SUSAR was associated with lethal outcome (hepatic insufficiency, Doc-C arm). In PlanB, six treatment-related deaths were reported [10]. Overall, grade 3/4 adverse events were observed significantly more often in patients receiving anthracycline-containing chemotherapy than those receiving anthracycline-free chemotherapy (76.3% vs. 70.1%; *P* < 0.001). More specifically, patients in the anthracycline-containing chemotherapy arm had significantly more leukopenia grade 3/4, nausea grade 3/4, fatigue grade 3/4, vomiting grade 3/4 and stomatitis grade 3/4 than patients in the anthracycline-free chemotherapy arm (see Table 2A). In contrast, there was no specific adverse event that occurred significantly more often in the anthracycline-free chemotherapy arm. The frequency of antibiotic treatment did not differ significantly between the two chemotherapy arms, but G-CSF treatment was applied significantly more often in the anthracycline-containing chemotherapy arm (Table 2B).

**Table 2:** (A) Frequency (number of patients) of grade 3/4 adverse events (overall, most common adverse events; CTCAE V 3.0) according to chemotherapy arm (anthracycline-containing vs. anthracycline-free); (B) antibiotic treatment and G-CSF treatment according to chemotherapy arm (anthracycline-containing vs. anthracycline-free).

Variable	Anthracycline-containing chemotherapy (FEC-Doc^a^/EC-Doc^b^; *N* = 2944)	Anthracycline-free chemotherapy (Doc-C^c^; *N* = 2980)	*P* value^d^
**(A)**			
Any adverse event	2245 (76.3%)	2089 (70.1%)	<0.001*
Anaemia	20 (0.7%)	21 (0.7%)	0.91
Leukopenia	1509 (51.3%)	1358 (45.6%)	<0.001*
Neutropenia	1187 (40.3%)	1101 (36.9%)	0.008
Nausea	88 (3.0%)	40 (1.3%)	<0.001*
Fatigue	131 (4.4%)	83 (2.8%)	0.001*
Vomitting	53 (1.8%)	18 (0.6%)	<0.001*
Stomatitis	57 (1.9%)	26 (0.9%)	<0.001*
Constipation	21 (0.7%)	12 (0.4%)	0.11
Diarrhoea	55 (1.9%)	63 (2.1%)	0.50
SGPT elevation	46 (1.6%)	39 (1.3%)	0.41
SGOT elevation	10 (0.3%)	6 (0.2%)	0.31
Pain	68 (2.3%)	45 (1.5%)	0.024
Infection	59 (2.0%)	78 (2.6%)	0.12
Neuropathy	45 (1.5%)	23 (0.8%)	0.006
Arthralgia	45 (1.5%)	29 (1.0%)	0.054
Febrile neutropenia	114 (3.9%)	145 (4.9%)	0.062
**(B)**			
Antibiotic treatment			0.16
No	2217 (75.3%)	2288 (76.8%)	
Yes	726 (24.7%)	688 (23.1%)	
Unknown	1 (0.0%)	4 (0.1%)	
G-CSF treatment			0.001*
No	1598 (54.3%)	1734 (58.2%)	
Yes	1285 (43.6%)	1162 (39.0%)	
Unknown	61 (2.1%)	84 (2.8%)	

Data pooled from the Success C and PlanB trials.

^a^FEC-Doc: 3× fluorouracil_500_-epirubicin_100_-cyclophosphamide_500_ q3w followed by 3× docetaxel_100_ q3w.

^b^EC-Doc: 4× epirubicin_90_-cyclophosphamide_600_ q3w followed by 4× docetaxel_100_ q3w.

^c^Doc-C: 6× docetaxel_75_-cyclophosphamide_600_ q3w.

^d^Chi-square test.

*Significant after significance level was adjusted for multiple comparisons using Bonferroni correction.

## Discussion

Based on the results of our pooled analysis with individual data from almost 6000 patients, we found that six cycles of TC (TC6) provide similar efficacy compared to an anthracycline-containing regimen in most patients with HER2-negative early breast cancer and showed significantly lower incidence of overall grade 3/4 toxicities. However, subgroup analyses indicate that patients with a high tumour burden in terms of four or more involved lymph nodes and patients with lobular tumours might benefit from anthracycline-containing chemotherapy, at least in terms of increased DFS. A more detailed analysis incorporating two-way and three-way interaction tests showed that this benefit may be limited to the subset of patients with pN2/3 lobular tumours only. These findings are in contrast to the published analysis by Blum et al. from the ABC trials (*n* = 4242 patients), which could not demonstrate non-inferiority of anthracycline-free therapy (TC6) compared to anthracycline-containing regimens with regard to invasive DFS in patients with high-risk HER2-negative early breast cancer [9]. The three adjuvant trials included (US Oncology Research (USOR) 06-090, National Surgical Adjuvant Breast and Bowel Project (NSABP) B-46-I/USOR 07132 and NSABP B-49) mostly used concomitant taxanes (docetaxel, doxorubicin and cyclophosphamide—TAC chemotherapy regime); only in the NSABP B-49 trial investigators could choose both between concomitant or sequential taxanes and between paclitaxel or docetaxel.

The impact of different available agents (docetaxel, paclitaxel, nab-paclitaxel) and taxane schedules (concomitant or sequential) within anthracycline/taxane-chemotherapies on outcome in early breast cancer patients has been controversially discussed. DFS in high-risk breast cancer patients, treated with anthracycline-containing chemotherapy, is improved with weekly paclitaxel (odds ratio (OR) for DFS 1.27; *P* = 0.006) and also docetaxel every 3 weeks (OR 1.23; *P* = 0.02) compared to paclitaxel every 3 weeks [18]. With regard to taxane schedules, two large trials revealed no difference in efficacy between concomitant versus sequential anthracycline/taxane-based chemotherapy regimens [16, 17]. In contrast, a study from Swain et al. from 2010 showed a DFS benefit with sequential AC-T compared to doxorubicin-docetaxel (AT) or concurrent AC-T and, in addition, also an improved OS with sequential AC-T compared to AT [18]. Similarly, Oakmen et al. found an improvement of OS after 8 years in node-positive early breast cancer patients treated with sequential docetaxel compared to concurrent doxorubicin-docetaxel [19]. A meta-analysis of three Phase III trials likewise showed a significant improvement of DFS and OS with sequential versus concurrent anthracycline/taxane-containing chemotherapy in adjuvant treatment of early breast cancer patients (DFS: RR 0.90, 95% CI: 0.84–0.98, *P* = 0.01; OS: RR 0.88, 95% CI: 0.79–0.98, *P* = 0.02) [20]. These findings indicating a survival benefit of sequential versus concurrent anthracycline/taxane-containing chemotherapy might be related to the fact that the cumulative dose of taxanes usually is higher for sequential than simultaneous taxane application within an anthracycline/taxane-chemotherapy regime. In addition, the use of sequential taxanes in anthracycline/taxane chemotherapy often leads to prolonged treatment duration which might also influence treatment efficacy.

These points could explain the discordant conclusion of PlanB/Success C compared to the ABC trials where mostly TAC with simultaneous taxanes was used as a chemotherapy regime. In addition, median follow-up time in the ABC trial was 3.3 years with only 2.2 years in the largest of the three included trials (NSABP B-49) compared to 62 months (5.16 years) in our pooled analysis. Longer follow-up is especially important in HR + disease, given the frequent occurrence of late recurrences (after 5 years) in this subset of early breast cancer patients [21]. In addition, no benefit for any chemotherapy regime in terms of increased iDFS was seen in the subset of patients with pN0 and the patients treated within the largest trial NSABP B-49 (*n* = 1819).

Though the used taxane dose per cycle is lower (but cumulative taxane dose is somewhat higher) in the anthracycline-free TC6 regime (docetaxel 75 mg/m² every three weeks) compared to anthracycline-containing regimes with sequential taxane use (docetaxel 100 mg/m² every three weeks), TC6 showed comparable efficacy in the included patients. However, according to our results, especially the subset of patients with pN2/pN3 lobular tumours seems to profit from anthracycline-containing chemotherapy. This particular subgroup showed not only a clearly improved DFS but also better OS with anthracyclines. While there is evidence that high-risk lobular breast cancers clearly benefit from chemotherapy [22], detailed knowledge regarding particular sensitivity to anthracycline-containing vs anthracycline-free chemotherapy in different histological breast cancer subtypes is currently not available. Several markers have been postulated to indicate sensitivity to anthracycline-containing chemotherapy including topoisomerase II alpha (TOP2A) as the most promising one, but—as pointed out by a recent review—results of retrospective analyses are mostly inconsistent and difficult to interpret, and confirmation from prospectively randomised studies that TOP2A amplification is associated with increased sensitivity to anthracyclines is still lacking [23].

A significant two-way interaction between chemotherapy arm and nodal stage for patients with HR-positive disease was reported by Blum et al. [9]. Whereas the analysis of Blum et al. also suggested potential benefits of A-containing chemotherapy for patients with HR-negative tumours (triple-negative) [24], our data do not support this finding. Based on our results, patients with triple-negative tumours do not seem to benefit from additional treatment with anthracyclines. Results from the neoadjuvant setting suggest that they may gain further benefit by the addition of platinum to anthracycline-taxane chemotherapy [25, 26]. Nevertheless, the anthracycline-free combination of docetaxel and carboplatin has also shown high efficacy in neoadjuvant triple-negative breast cancer [27] which was even comparable to that of an anthracycline-taxane and platinum sequence in the NeoSTOP trial [28]. Recent data from an adjuvant Phase III trial in triple-negative early breast cancer even showed improved DFS of six cycles carboplatin-paclitaxel compared to 3× FEC and 3× DOC [29]. In addition, our results indicate that anthracycline-free chemotherapy is also a valid option for younger patients (<40 years) or patients with larger tumours (pT3/pT4).

The restriction of “taxane use” in the A-containing treatment arm to sequential docetaxel 100 mg/m² every 3 weeks for three or four cycles only is a major strength of the presented pooled analysis and prevents potential bias through different taxane regimes, agents and application intervals. However, a limiting factor is that the standard treatment arm of this pooled analysis includes two different AC-T chemotherapy regimens with different duration of chemotherapy exposure consisting of either 3 × FEC/3 × DOC (Success C) or 4 × EC/4 × DOC (PlanB). In addition, though FEC-DOC is an effective regimen, 5-FU is not considered standard anymore in this setting, as the addition of 5-FU to an anthracycline/cyclophosphamide combination failed to show any relevant benefit in improvement of DFS [30]. However, multivariable testing was adjusted for the different treatment regimens and thereby, the interaction results are not biased by a “trial-effect”. Another limitation is the fact that—despite the large overall sample size of this pooled analysis—there is still a 20% risk to show a false negative result with regard to the detection of a 2% difference in 5-year DFS (as the retrospectively calculated power was 80%), and that some of the subgroups analysed were still rather small with a corresponding further loss of statistical power for the post hoc comparisons involving these subgroups. Lastly, median follow-up in our pooled analysis comprises only 5 years and thus late disease recurrences might have been missed.

Limiting the incorporation of anthracyclines only for selected patients is desirable to prevent avoidable toxicities without a gain in efficacy. Our study represents currently the largest for assessing the impact of the addition of anthracyclines to a taxane-based regimen in intermediate to high-risk HER2-negative early breast cancer. The presented pooled analysis clearly addresses the question of TC vs AC-T therapy in adjuvant breast cancer treatment. The data suggest that six cycles of TC represent an effective chemotherapy option with less toxicity for HER2-negative early breast cancer and based on these results the indication for TC6 could be expanded towards intermediate-risk early breast cancer patients. Exploratory subgroup analyses indicated that some high-risk patients may benefit from anthracycline-containing chemotherapy in terms of improved DFS but not OS. Further evaluation of those results by additional interaction analyses showed no benefit for the majority of patients. Only the subgroup of lobular-invasive pN2/pN3 early breast cancer-derived significant benefit regarding better DFS and OS with the addition of anthracycline. Thus, a clear benefit of anthracycline-containing chemotherapy is observed only for about 2% of the overall HER2-negative early breast cancer study population in the here presented analysis (acknowledging that in the rest of the population this pooled analysis has only 80% power to detect a 2% difference in 5-year DFS). Suggesting that this specific subgroup may have different biological features, we need further information for a better understanding of this subset of patients. Our results indicate that the simple classification of patient groups only according to isolated factors like “node-positive” or “histological type” could be insufficient to identify patient subgroups that might truly benefit from anthracyclines. The impact of anthracyclines has to be addressed in future randomised trials specifically aimed at selected high-risk breast cancer patients.

## Supplementary information

Supplemental Tables

Supplemental Figures

Supplemental Material

AJ Checklist

Consort Checklist

## Data Availability

De-identified data will be made available to other researchers, subject to the approval of a formal data access request that includes a detailed description of the purpose/scientific rationale of the proposed project. Requests are reviewed by the study groups Steering Committees and will be approved if the proposed projects have a sound scientific or patient benefit rationale. Data recipients are required to sign a formal data sharing agreement that describes the conditions for release and requirements for data transfer, storage, archiving, publication and intellectual property. Data will be available beginning 9 months and ending 5 years following article publication. Additional documents (e.g., full study protocol, informed consent form) are available on request.
