# Supplementary material for: The impact of anthracyclines in intermediate and high-risk HER2-negative early breast cancer—a pooled analysis of the randomised clinical trials PlanB and SUCCESS C
Source: Br J Cancer. 2022 Feb 22;126(12):1715–24. doi: 10.1038/s41416-021-01690-6 (PMC9174181; doi:10.1038/s41416-021-01690-6)
Supplement: Supplementary file 1 — Supplemental Tables [file 41416_2021_1690_MOESM1_ESM.docx]

**Table S1:** Baseline and clinic-pathological characteristics of patients in PlanB and SUCCESS C

| Variable | Total N = 5924 | Success C N = 3643 | PlanB N = 2281 | p-value^1^ |
| --- | --- | --- | --- | --- |
| Age (years)  *Median*  *Range* | 55.0  24 - 79 | 55.0  24 - 79 | 55.0  25 - 77 | 0.474^2^ |
| Menopausal status  *premenopausal*  *postmenopausal*  *unknown* | 2249 (38.0)  3503 (59.1%)  172 (2.9%) | 1436 (39.4%)  2207 (60.6%)  0 (0.0%) | 813 (35.6%)  1296 (56.8%)  172 (7.5%) | 0.515^3^ |
| Tumor stage  *pT1*  *pT2*  *pT3*  *pT4* | 2857 (48.2%)  2753 (46.5%)  251 (4.2%)  63 (1.1%) | 1603 (44.0%)  1818 (49.9%)  174 (4.8%)  48 (1.3%) | 1254 (55.0%)  935 (41.0%)  77 (3.4%)  15 (0.7%) | < 0.001^4^ |
| Nodal stage  *pN0*  *pN1*  *pN2*  *pN3*  *unknown* | 2859 (48.3%)  2485 (41.9%)  430 (7.3%)  149 (2.5%)  1 (0.0%) | 1530 (42.0%)  1702 (46.7%)  301 (8.3%)  109 (3.0%)  1 (0.0%) | 1329 (58.3%)  783 (34.3%)  129 (5.7%)  40 (1.8%)  0 (0.0%) | < 0.001^4^ |
| Histological grading  *G1*  *G2*  *G3*  *unknown* | 369 (6.2%)  3167 (53.5%)  2384 (40.2%)  4 (0.1%) | 232 (6.4%)  1857 (51.0%)  1554 (42.7%)  0 (0.0%) | 137 (6.0%)  1310 (57.4%)  830 (36.4%)  4 (0.2%) | < 0.001^4^ |
| Histological type  *ductal*  *lobular*  *other* | 4808 (81.2%)  726 (12.3%)  390 (6.6%) | 2930 (80.4%)  507 (13.9%)  206 (5.7%) | 1878 (82.3%)  219(9.6%)  184 (8.1%) | < 0.001^3^ |
| Hormone receptor status  *negative*  *positive* | 1279 (21.6%)  4645 (78.4%) | 859 (23.6%)  2784 (76.4%) | 420 (18.4%)  1861 (80.6%) | < 0.001^3^ |
| Type of surgery  *breast conserving*  *mastectomy*  *other* | 4642 (78.4%)  1141 (19.3%)  141 (2.4%) | 2782 (76.4%)  720 (19.8%)  141 (3.9%) | 1860 (81.5%)  421 (18.5%)  0 (0.0%) | < 0.001^3^ |
| Biological subtype  *luminal A like*  *luminal B like*  *triple negative*  *unknown* | 3323 (56.1%)  1319 (22.3%)  1279 (21.6%)  3 (0.1%) | 1946 (53.4%)  838 (23.0%)  859 (23.6%)  0 (0.0%) | 1377 (60.4%)  481 (21.1%)  420 (18.4%)  3 (0.1%) | < 0.001^3^ |
| Adjuvant chemotherapy  *anthracycline-containing^5^*  *anthracycline-free^6^* | 2944 (49.7%)  2980 (50.3%) | 1816 (49.8%)  1827 (50.2%) | 1128 (49.5%)  1153 (50.5%) | 0.766^3^ |
| Radiotherapy  *no*  *yes*  *unknown* | 1139 (19.2%)  4780 (80.7%)  5 (0.1%) | 533 (14.6%)  3110 (85.4%)  0 (0.0%) | 606 (26.6%)  1670 (73.2%)  5 (0.2%) | < 0.001^3^ |

^1^ all tests without unknowns
^2^ Mann-Whitney U test
^3^ Chi-square test
^4^ Cochran-Armitage test for trend
^5^ Success C: FEC-Doc; 3 x fluorouracil_500_-epirubicin_100_-cyclophosphamide_500_ q3w followed by 3 x docetaxel_100_ q3w; PlanB: EC-Doc; 4 x epirubicin_90_-cyclophosphamide_600_ q3w followed by 4 x docetaxel_100_ q3w
^6^ Success C and PlanB: Doc-C; 6 x docetaxel_75_-cyclophosphamide_600_ q3w

**Table S2.** Results of a multivariable cox regression (proportional hazards model) for disease-free survival including all main effects.

| **Variable** | **Hazard ratio** | **95% CI** | **P- value** |
| --- | --- | --- | --- |
| Chemotherapy arm  *anthracycline-free vs. anthracycline-containing* | 1.010 | 0.856 – 1.192 | 0.908 |
| Study  *PlanB vs. Success C* | 1.235 | 1.037 – 1.470 | 0.018 |
| Age class  *41 - 60 vs. ≤ 40*  *> 60 vs. ≤ 40* | 0.958  1.210 | 0.680 – 1.350  0.810 – 1.810 | 0.096  0.807  0.352 |
| Menopausal status  *postmenopausal vs. premenopausal* | 0.869 | 0.695 – 1.086 | 0.217 |
| Type of surgery  *mastectomy vs. breast conserving*  *other vs. breast conserving* | 1.453  0.882 | 1.181 – 1.789  0.482 – 1.615 | 0.001  < 0.001  0.684 |
| Tumor stage  *pT2 vs. pT1*  *pT3/pT4 vs. pT1* | 1.585  1.774 | 1.322 – 1.900  1.259 – 2.500 | < 0.001  < 0.001  0.001 |
| Nodal stage  *pN2/pN3 vs. pN0/pN1* | 2.653 | 2.138 – 3.293 | < 0.001 |
| Hormone receptor status  *positive vs. negative* | 0.476 | 0.388 – 0.583 | < 0.001 |
| Histological grading  *G2 vs. G1*  *G3 vs. G1* | 1.899  3.380 | 1.060 – 3.400  1.880 – 6.077 | < 0.001  0.031  < 0.001 |
| Histological type  *lobular vs. ductal*  *other vs. ductal* | 0.875  0.949 | 0.653 – 1.173  0.690 – 1.305 | 0.649  0.373  0.746 |

**Table S3:** First invasive disease-free survival events according to chemotherapy arm

| Type of event | Total N = 5924 | Anthracycline-containing chemotherapy N = 2944 | Anthracycline-free chemotherapy N = 2980 |
| --- | --- | --- | --- |
| *Locoregional recurrence*  *Distant recurrence*  *Site of first recurrence unknown*  *Death*  *Total* | 93  379  27  82  581 | 48  183  11  41  283 | 45  196  16  41  298 |

**Table S4**. Results of 2-way interactions between chemotherapy treatment arm and both nodal status and histological type, and the 3-way interaction between chemotherapy arm, nodal status and histological type.

| **Variable** | **P- value** |
| --- | --- |
| 2-way interactions  *chemotherapy arm * nodal status*  *chemotherapy arm * histological type* | 0.688  0.746 |
| 3-way interaction  *chemotherapy arm * nodal status * histological type* | 0.028 |
