# Supplementary material for: The impact of anthracyclines in intermediate and high-risk HER2-negative early breast cancer—a pooled analysis of the randomised clinical trials PlanB and SUCCESS C
Source: Br J Cancer. 2022 Feb 22;126(12):1715–24. doi: 10.1038/s41416-021-01690-6 (PMC9174181; doi:10.1038/s41416-021-01690-6)
Supplement: Supplementary file 2 — Supplemental Figures [file 41416_2021_1690_MOESM2_ESM.pptx]

## Slide 1
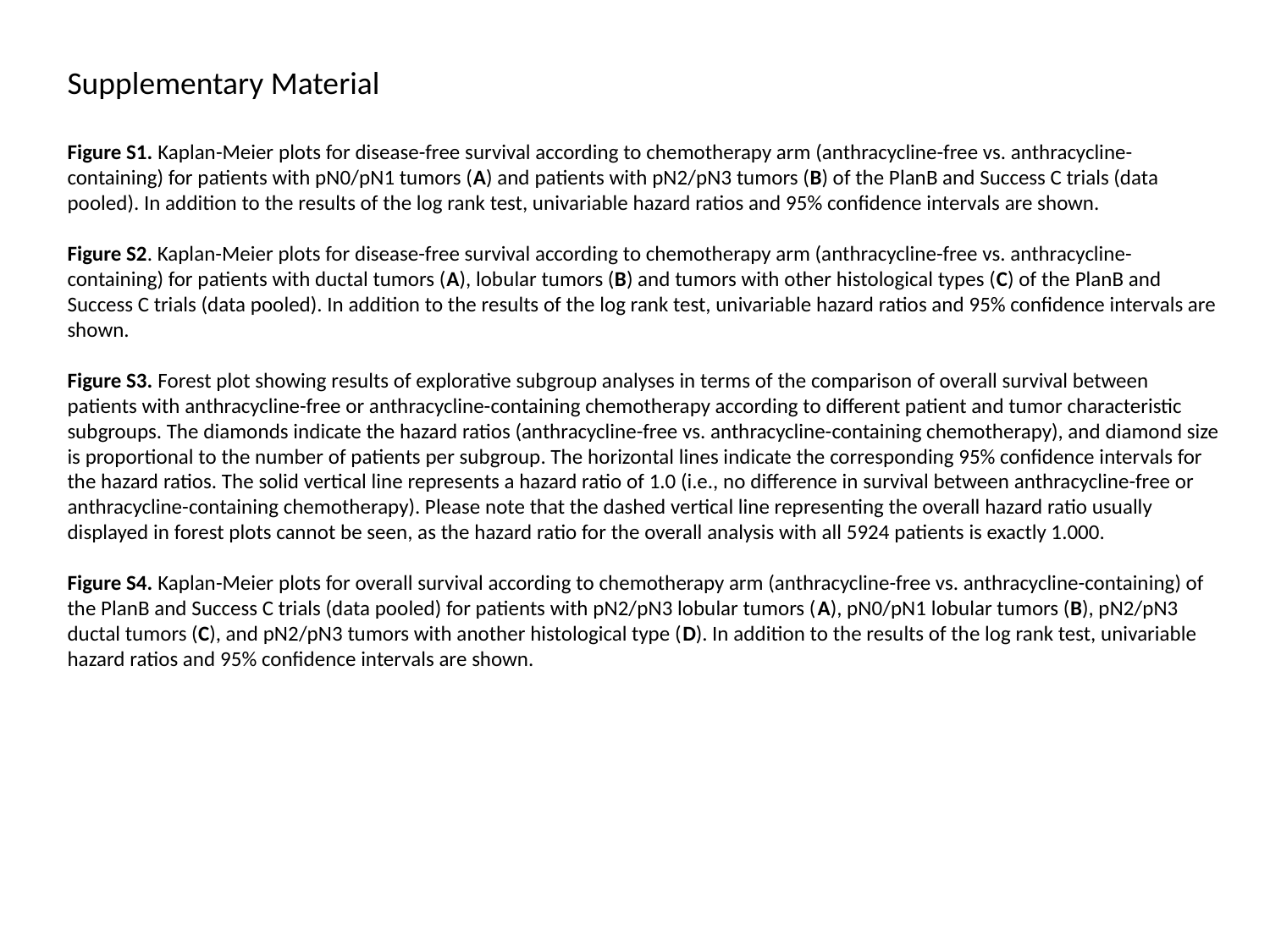

Supplementary Material
Figure S1. Kaplan-Meier plots for disease-free survival according to chemotherapy arm (anthracycline-free vs. anthracycline-containing) for patients with pN0/pN1 tumors (A) and patients with pN2/pN3 tumors (B) of the PlanB and Success C trials (data pooled). In addition to the results of the log rank test, univariable hazard ratios and 95% confidence intervals are shown.
Figure S2. Kaplan-Meier plots for disease-free survival according to chemotherapy arm (anthracycline-free vs. anthracycline-containing) for patients with ductal tumors (A), lobular tumors (B) and tumors with other histological types (C) of the PlanB and Success C trials (data pooled). In addition to the results of the log rank test, univariable hazard ratios and 95% confidence intervals are shown.
Figure S3. Forest plot showing results of explorative subgroup analyses in terms of the comparison of overall survival between patients with anthracycline-free or anthracycline-containing chemotherapy according to different patient and tumor characteristic subgroups. The diamonds indicate the hazard ratios (anthracycline-free vs. anthracycline-containing chemotherapy), and diamond size is proportional to the number of patients per subgroup. The horizontal lines indicate the corresponding 95% confidence intervals for the hazard ratios. The solid vertical line represents a hazard ratio of 1.0 (i.e., no difference in survival between anthracycline-free or anthracycline-containing chemotherapy). Please note that the dashed vertical line representing the overall hazard ratio usually displayed in forest plots cannot be seen, as the hazard ratio for the overall analysis with all 5924 patients is exactly 1.000.
Figure S4. Kaplan-Meier plots for overall survival according to chemotherapy arm (anthracycline-free vs. anthracycline-containing) of the PlanB and Success C trials (data pooled) for patients with pN2/pN3 lobular tumors (A), pN0/pN1 lobular tumors (B), pN2/pN3 ductal tumors (C), and pN2/pN3 tumors with another histological type (D). In addition to the results of the log rank test, univariable hazard ratios and 95% confidence intervals are shown.

## Slide 2
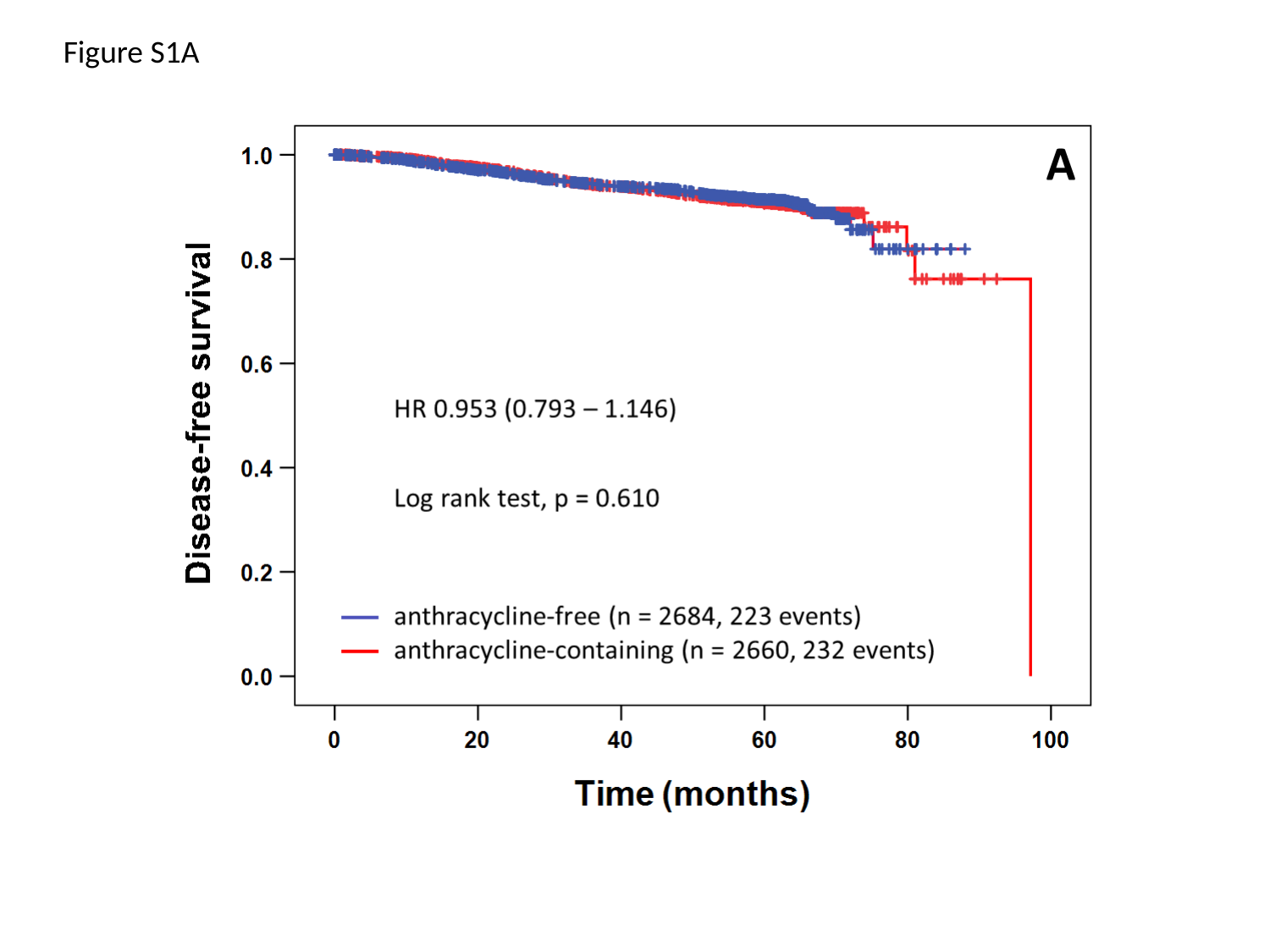

Figure S1A

## Slide 3
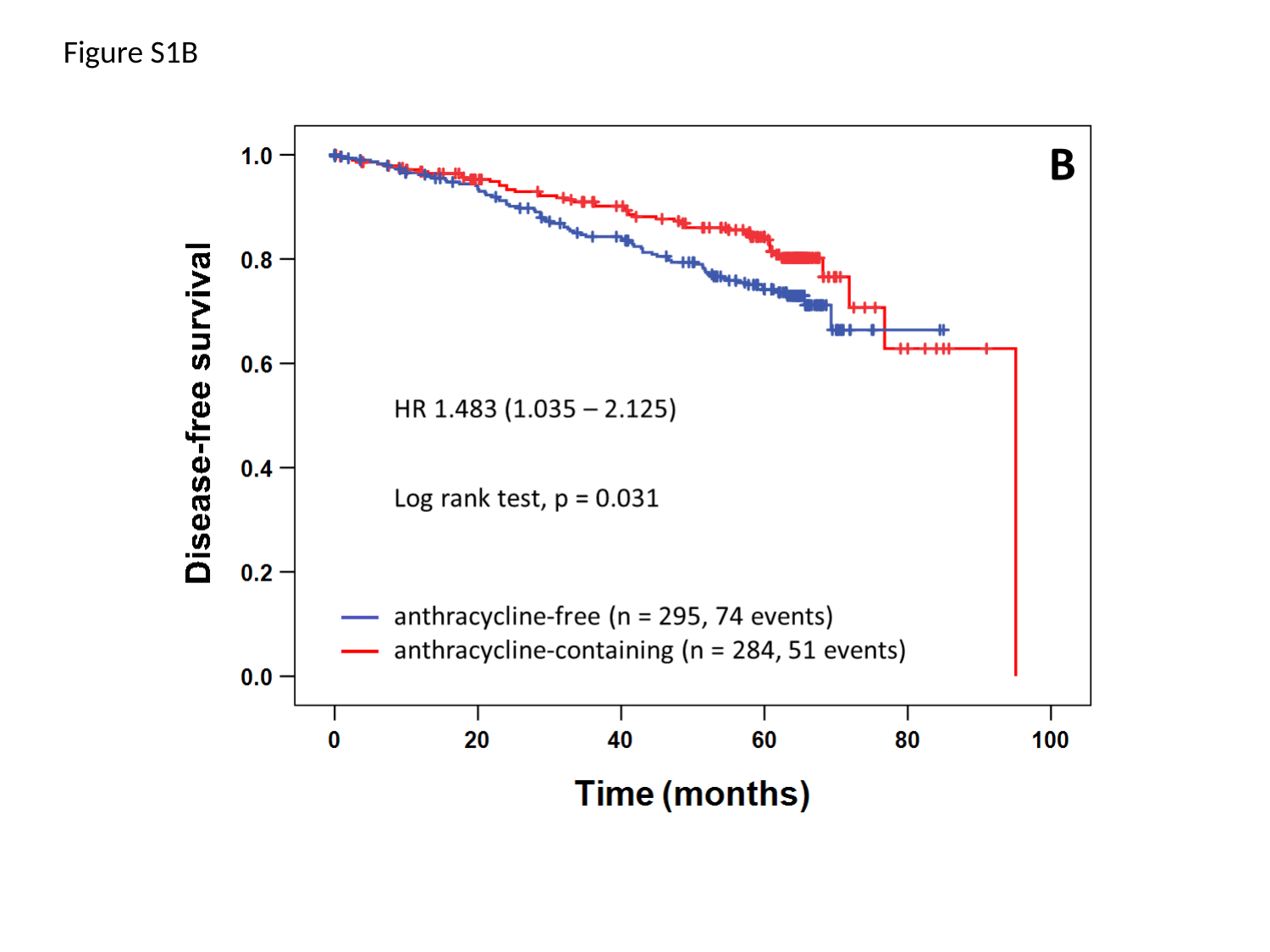

Figure S1B

## Slide 4
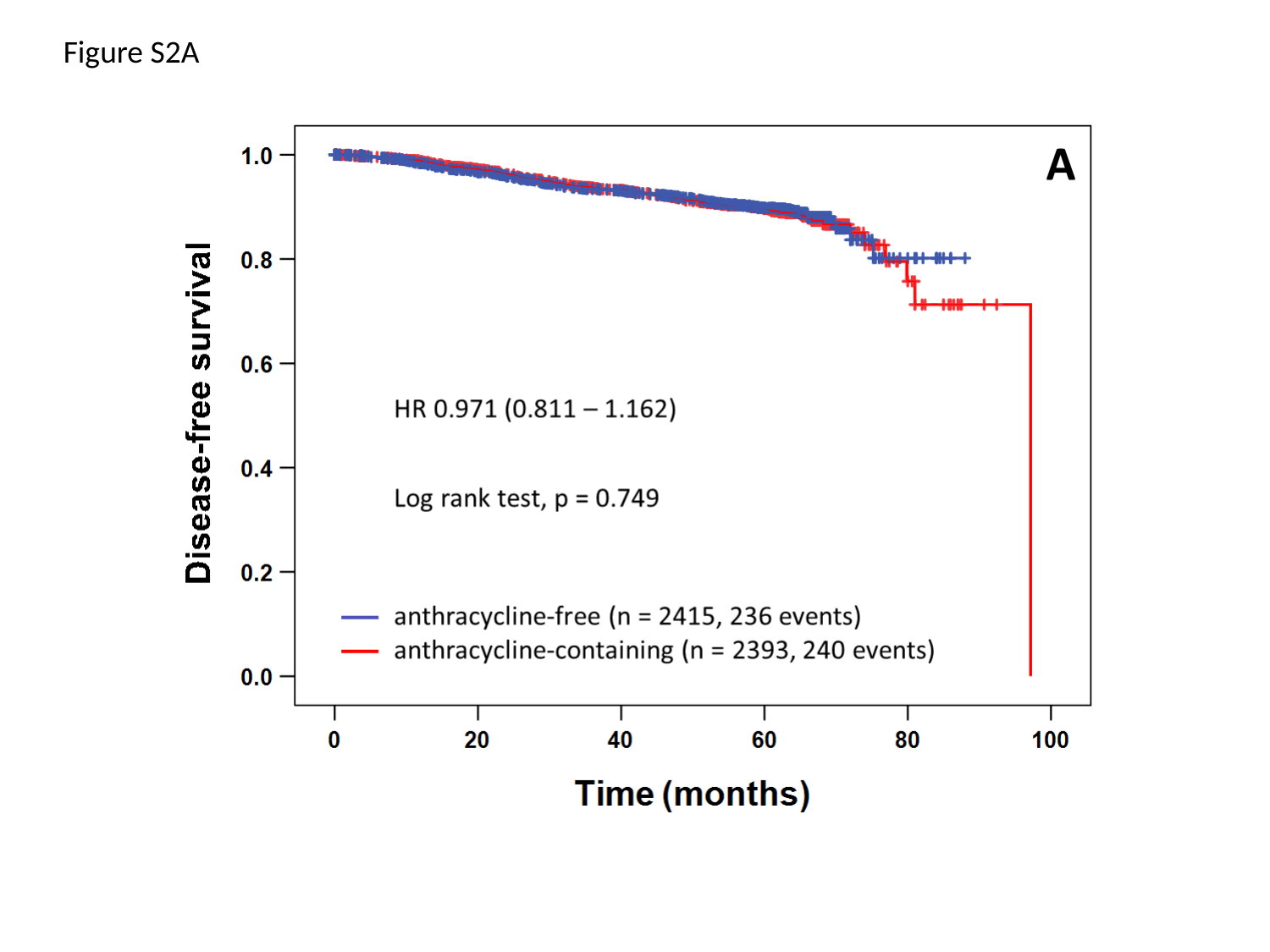

Figure S2A

## Slide 5
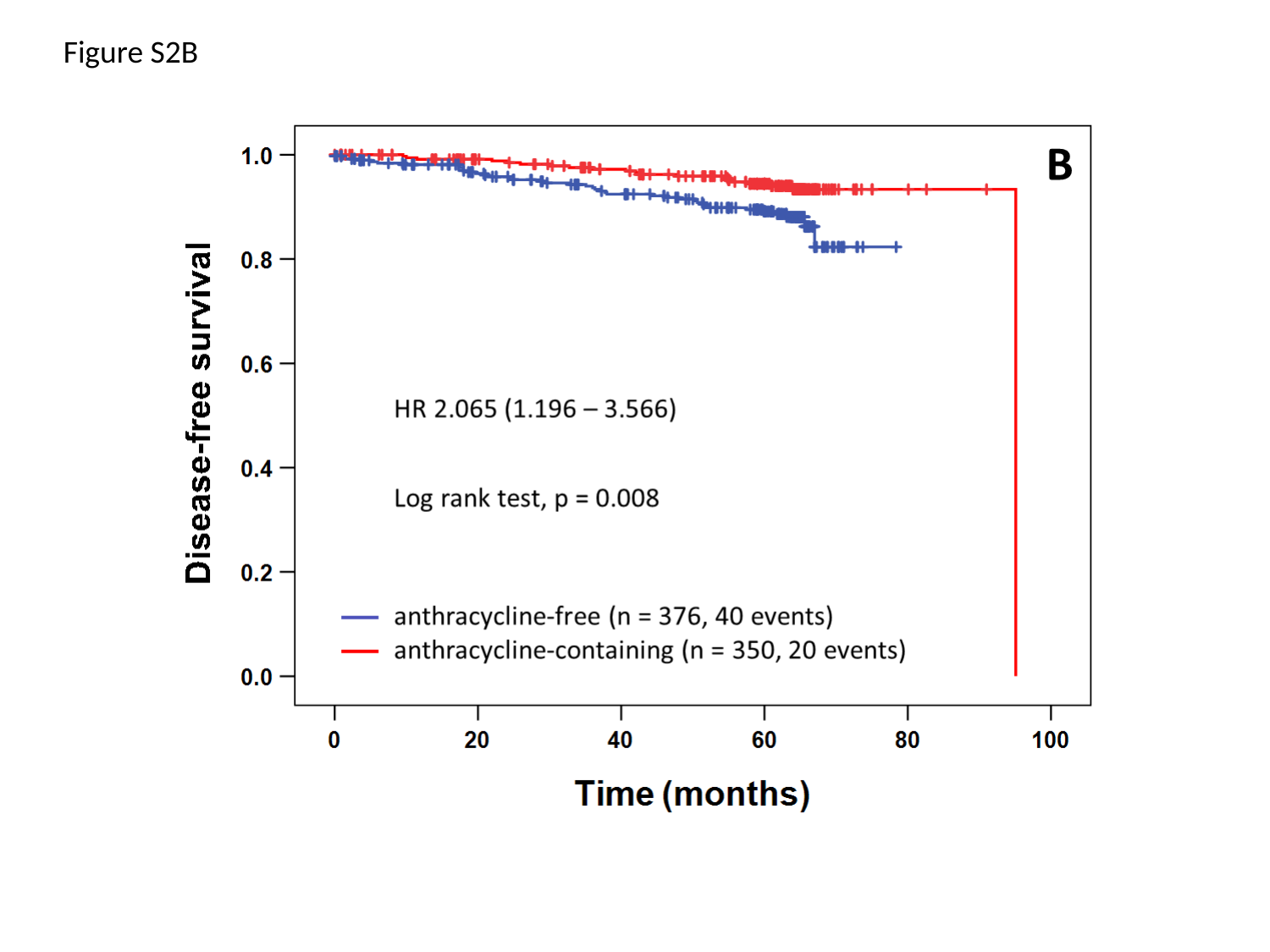

Figure S2B

## Slide 6
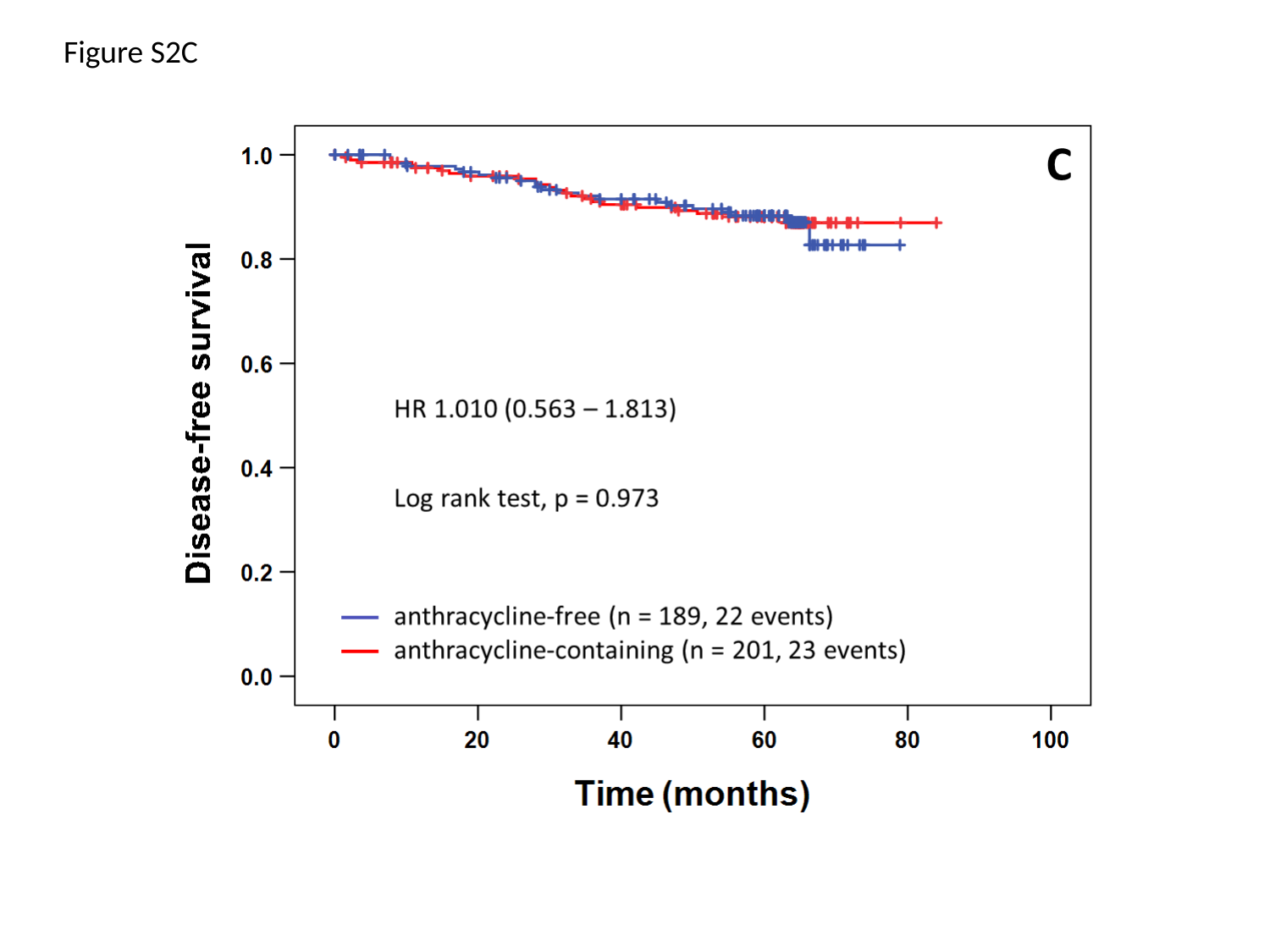

Figure S2C

## Slide 7
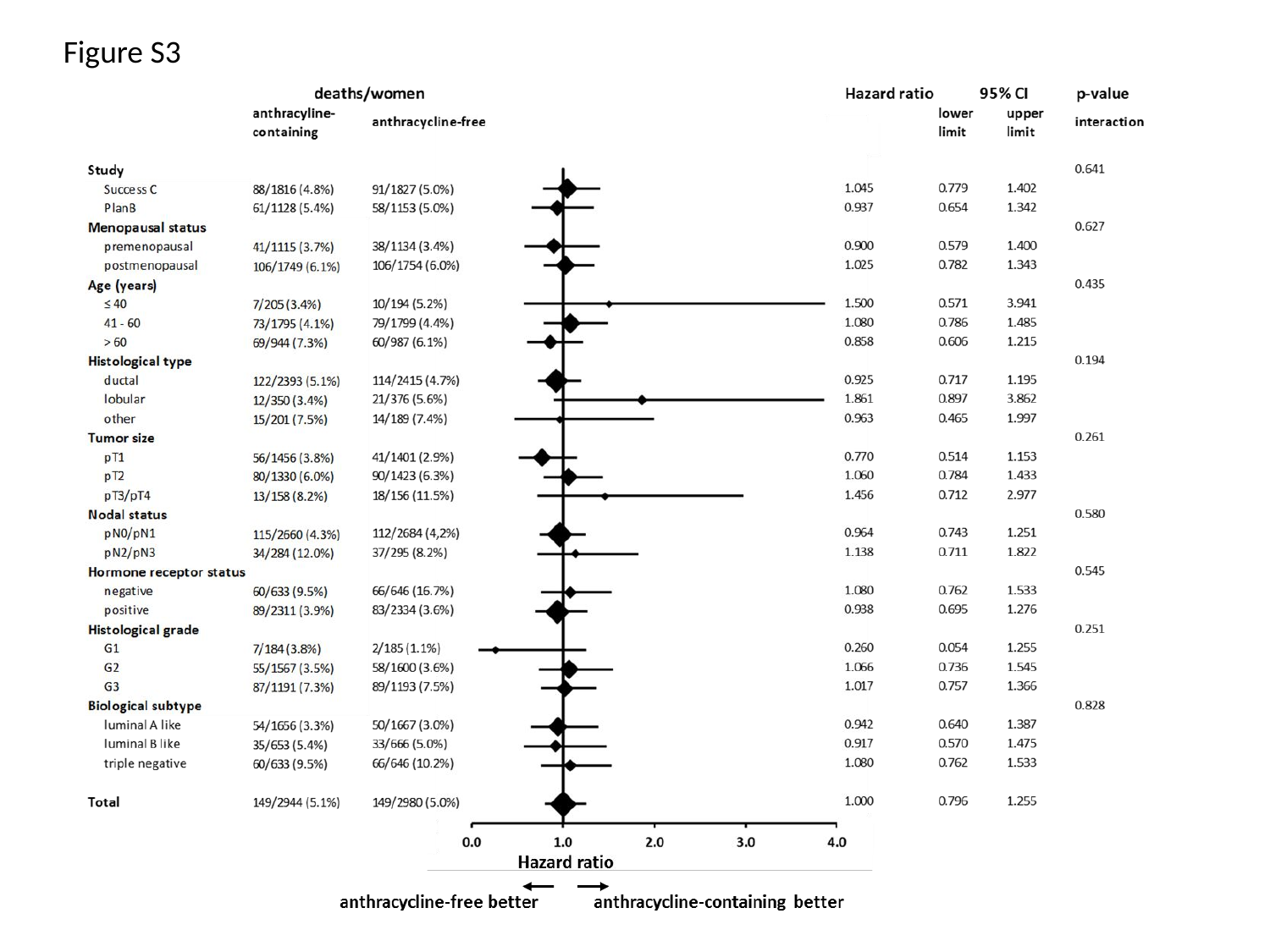

Figure S3

## Slide 8
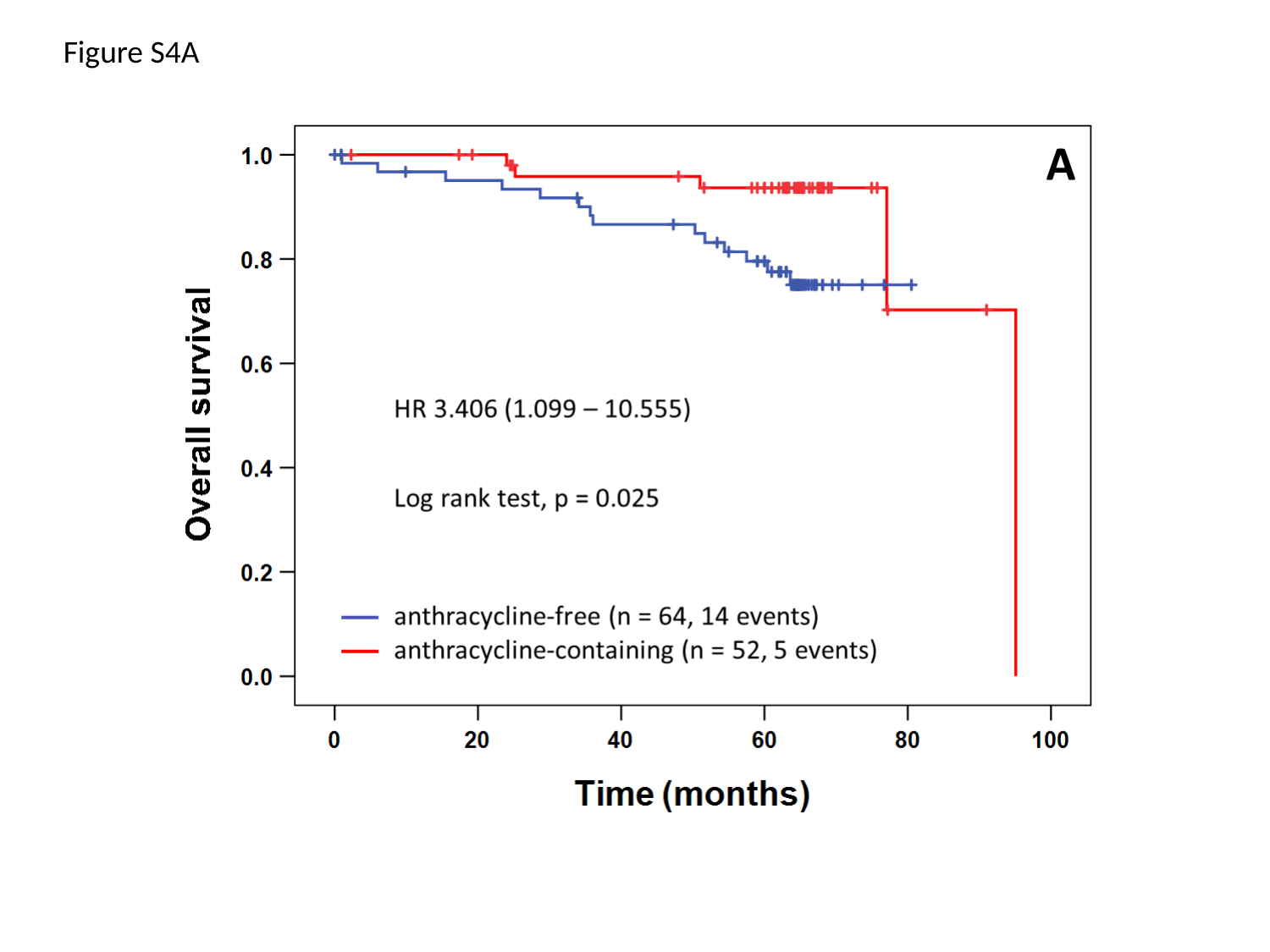

Figure S4A

## Slide 9
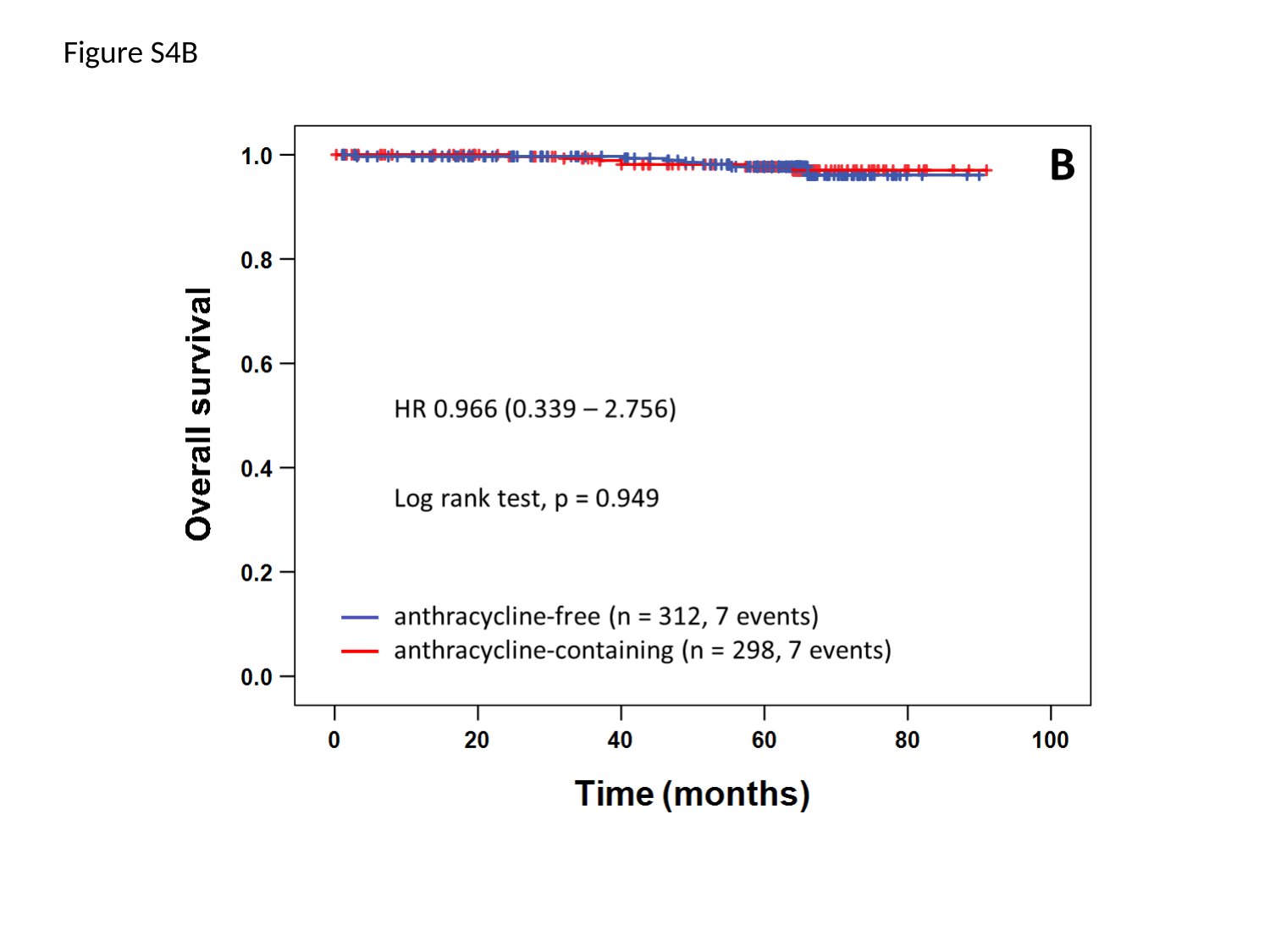

Figure S4B

## Slide 10
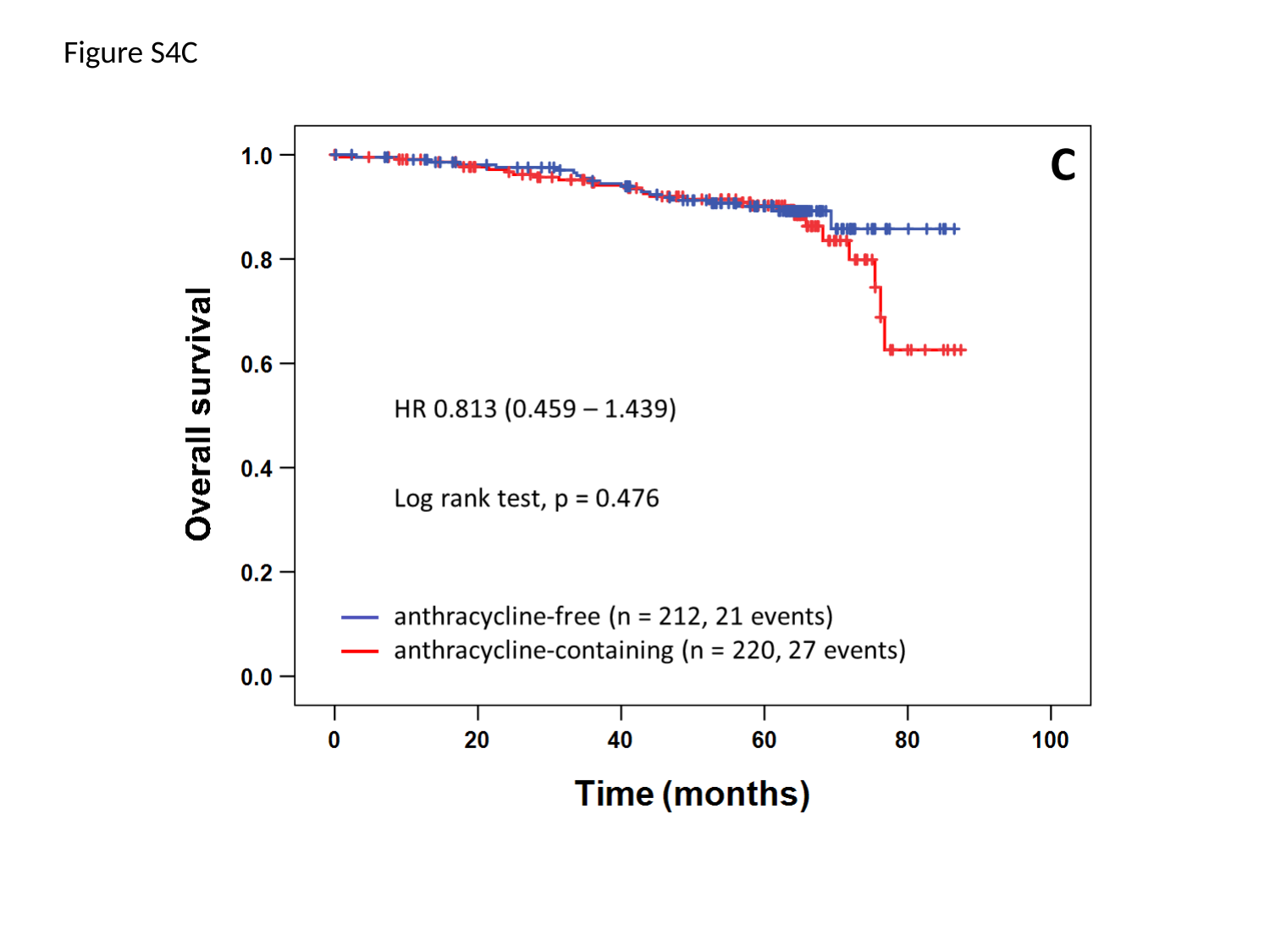

Figure S4C

## Slide 11
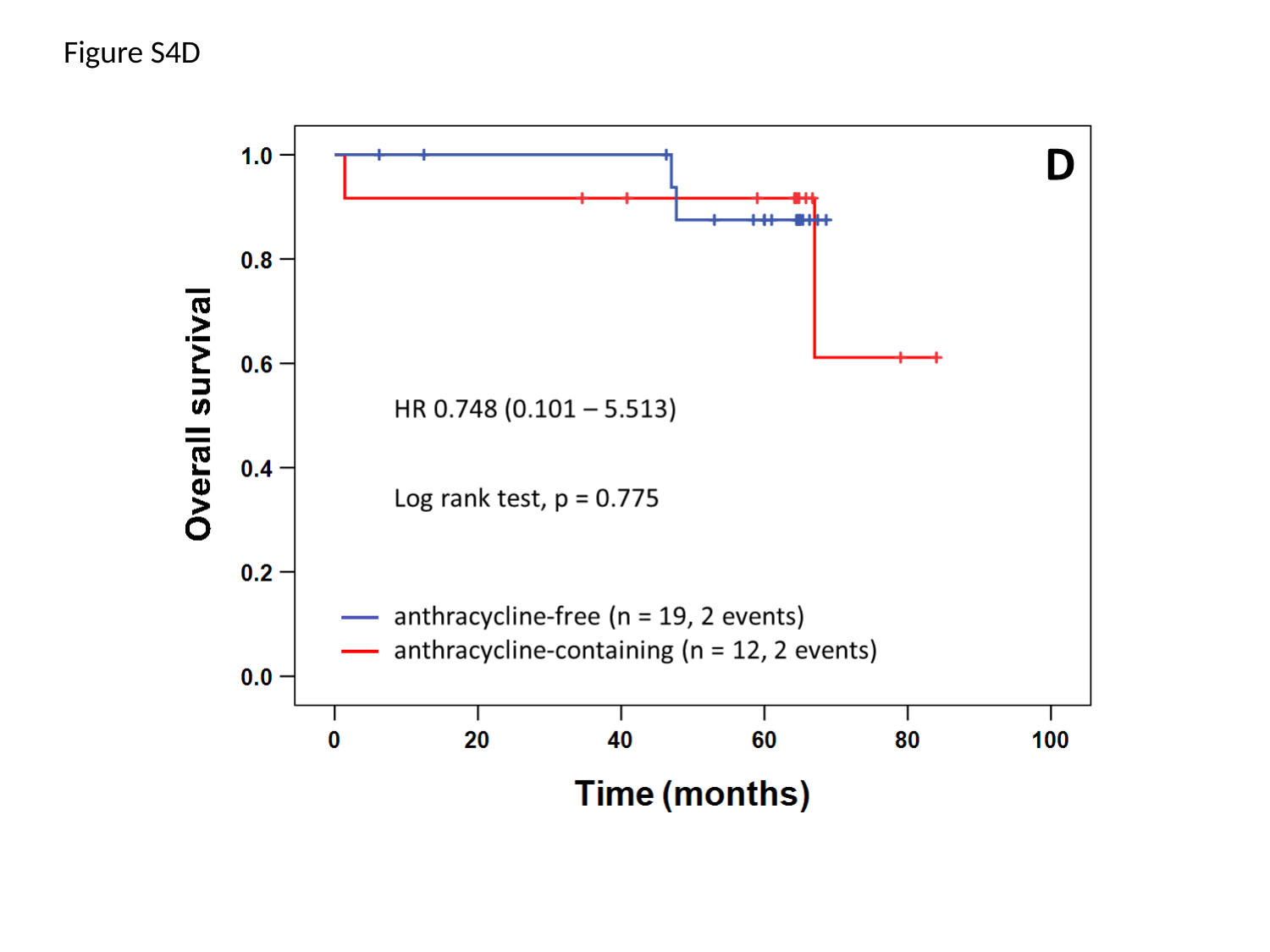

Figure S4D
