# Supplementary material for: The impact of anthracyclines in intermediate and high-risk HER2-negative early breast cancer—a pooled analysis of the randomised clinical trials PlanB and SUCCESS C
Source: Br J Cancer. 2022 Feb 22;126(12):1715–24. doi: 10.1038/s41416-021-01690-6 (PMC9174181; doi:10.1038/s41416-021-01690-6)
Supplement: Supplementary file 3 — Supplemental Material [file 41416_2021_1690_MOESM3_ESM.docx]

**Supplementary Material (Material S1):** Explorative analysis treatment by menopausal status in patients with lobular carcinomas.

We performed an exploratory analysis to investigate if the effect of anthracycline-containing chemotherapy observed in patients with lobular carcinomas was dependent on menopausal status. Postmenopausal patients with lobular carcinomas receiving the anthracycline-free chemotherapy (TC6) had significantly worse DFS compared to postmenopausal patients with lobular carcinomas receiving anthracycline- containing chemotherapy (n = 472; HR=2.82, 95% CI 1.39 – 5.73, p=0.003). No difference in DFS between the two chemotherapy regimens was found for premenopausal patients with lobular carcinomas (n = 241; HR=0.94, 95% CI 0.33 – 2.71, p=0.91). However, the 2-way interaction between chemotherapy treatment arm and menopausal status was not significant (p = 0.091); thus, the benefit of anthracycline-containing chemotherapy observed in patients with lobular carcinomas was not significantly affected by menopausal status.
